# Wnt5a Exhibits Layer-Specific Expression in Adult Skin, Is Upregulated in Psoriasis, and Synergizes with Type 1 Interferon

**DOI:** 10.1371/journal.pone.0005354

**Published:** 2009-04-28

**Authors:** Malgorzata Romanowska, Alan Evans, David Kellock, Susan E. Bray, Kathleen McLean, Susanne Donandt, John Foerster

**Affiliations:** 1 Division of Experimental Medicine, University of Dundee, Dundee, Scotland, United Kingdom; 2 Tayside Tissue Bank, University of Dundee, Dundee, Scotland, United Kingdom; 3 Department of Dermatology, University of Dundee, Dundee, Scotland, United Kingdom; 4 Charité Medical University, Berlin, Germany; Tulane National Primate Research Center, United States of America

## Abstract

**Background:**

Wnt5a is a member of the wingless-type patterning regulators important in pre-natal development. The expression and distribution of Wnt5a and its receptors frizzled (fzd) 3 and fzd 5 in adult human skin have not been comprehensively studied to date.

**Methodology/Principal Findings:**

We here show that Wnt5a, fzd3, fzd5, as well as fzd6 are restricted to specific layers in normal epidermis, analogous to their zonal distribution in hair follicles, suggesting a role in adult skin differentiation. In line, Wnt5a and fzd5 are both overexpressed and re-distributed in the epidermis of psoriasis which involves disturbed keratinocyte differentiation. Functionally, Wnt5a lowers the concentration of IFN required to induce target genes, and increases the magnitude of IFN target gene induction, suggesting a molecular mechanism underlying IFN hypersensitivity in psoriasis. Finally, we identify nedd8 and the amyloid precursor APP, previously shown to be upregulated in psoriasis, as targets of synergistic IFNα/Wnt5a induction.

**Conclusions/Significance:**

The present data (i) suggest that Wnt5a regulates epidermal differentiation even in adult skin and (ii) identify synergistic induction of type 1 IFN target genes as a novel mode of Wnt5a action. Targeting Wnt5a in the skin may reduce IFN hypersensitivity and be of therapeutical value.

## Introduction

Wnt proteins regulate diverse biological processes. Wnt5a represents the prototypical so-called non-canonical Wnt family members which signal not by inducing, or may even inhibit, the transcriptional activation of β-catenin. Apart from its complex role in development, Wnt5a is expressed in various adult tissues. Among its diverse biological activities, Wnt5a promotes proliferation in endothelial cells and glioblastoma cells [1]–[3], as well as adhesion in fibroblasts and breast cancer cells [4], [5]. Most recently, evidence has accumulated for the involvement of Wnt5a in inflammatory responses and innate immunity. Thus, Wnt5a is induced by Toll-like receptors and elicits secretion of IL12 and TNFα in response to mycobacterial infection [6]. Furthermore, Wnt5a is drastically upregulated in macrophages by LPS and IFNγ and induces the production of the pro-inflammatory cytokines IL1, IL6, and IL8 [7]. Wnt5a is also upregulated in synovial fibroblasts of inflammatory joints in rheumatoid arthritis where it induces IL6, IL8, and IL15 [8].

The signal transduction of Wnt5a appears to be context-dependent [9]. In all of the inflammatory responses cited above, as well as in endothelial cell activation, the active receptor appears to be Frizzled (Fzd) 5 [10]. However, signalling through Fzd3, and Ror2 have been described [4], [9].

The epidermis represents a dynamic system of highly regulated keratinocyte stem-cell renewal, proliferation, differentiation, and migration. At the same time, the epidermis acts as first-line barrier in innate immunity and, as such, maintains complex communication with resident dendritic cells and sub-epidermal endothelial cells and fibroblasts. Although Wnt5a is expressed in all of these cell types and its role in the development of skin appendages including hair follicles [11] and mammary glands [12] has been examined, surprisingly little is known about the expression, distribution, and function of Wnt5a in post-natal human epidermis.

Psoriasis is a very common chronic inflammatory skin disease with a prevalence of 2–4% in Caucasian populations. In psoriasis, keratinocyte proliferation as well as differentiation are perturbed, leading to a grossly thickened epidermis with an altered composition of normal epidermal layers. Apart from keratinocyte turnover, psoriasis involves a hyperactive innate immune response. Thus, iatrogenic administration of type 1 IFN (IFNα or IFNβ) elicits disease flares in susceptible individuals, as does endogenous IFN release in the course of viral disease [13], [14]. Moreover, keratinocytes from psoriasis patients are hypersensitive to type 1 IFN [15] and epidermal dendritic cells produce IFNα leading to massive upregulation of type 1 IFN target genes [16]. Indeed, hypersensitivity towards type 1 IFN induced by deletion of the IFN repressor IRF2 is sufficient to cause a psoriasis-like skin disease in mice [17].

We and others have previously found Wnt5a to be upregulated on the mRNA level in psoriasis [16], [18]. Here, we show that Wnt5a and its putative receptors are localized to specific compartments in normal skin and that Wnt5a is both highly upregulated and re-distributed in the hyperproliferative epidermis in psoriasis. Functionally, we show that Wnt5a sensitizes keratinocytes towards type 1 IFN. Finally, we identify nedd8 and APP as novel targets of synergistic IFN/Wnt5a activation.

## Materials and Methods

### Immunohistochemistry

Paraffin-embedded samples were obtained from the Tayside Tissue bank. Prior to biopsy, patients gave written consent to storage and analysis of biopsy samples. Storage and use of all tissues included in the work presented here was approved by the Tayside Committee on Medical Research Ethics B (REC ref. Nr. 07/S1402/90). Antibodies used were anti wnt5a (R&D, order nr. AF645, final dilution 1∶400), anti-frizzled 3 (Insight Biotech, ordered through Acris Antibodies, Germany, order nr. SP4568P, 1∶200), anti-frizzled 5 (Cambridge Bioscience, ARP41245_P050, 1∶800), anti-frizzled 6 (GenWay, 18-461-10538, ordered through Acris Antibodies, Germany; 1∶400). A detailed protocol is given in Supporting Methods S1.

### Keratinocyte primary culture and western blot

All studies on human samples were done with prior approval of the local institional ethics committee. Processing of biopsy samples and primary keratinocyte culture was performed as described [16]. Cells were fractionated using the NE-PER kit (Pierce, order-nr 78833) and 20 µg of protein loaded per lane onto 15% acrylamide-SDS gels. Blotting was performed on a semi-dry blotter onto nitro-cellulose membranes (Schleicher & Schell, 10 402 594). Blots were blocked in Tris-buffered saline containing 0.1% Tween-20 (TBST) and incubated with primary antibodies in TBST containing 4% dry milk as follows: rabbit anti-human nedd8 (Cell Signaling, #2745) and rabbit anti-human Amyloid β precursor protein (Biozol, ab12269) at 1∶1000, respectively, overnight at 4°C. Secondary antibody was anti-rabbit-HRP (Amersham Biosciences, NA934) at 1∶2000 for 1 h at RT. Blots were developed with ECL Plus (Amersham #RPN2 132) and scanned with a CCD camera.

### Stimulation with Wnt5a-mimic peptide and IFNα

Formylated hexapeptide formyl-MDGCEL [5] was synthesized by Pepscan Systems, reconstituted to 10 mM in PBS, aliquoted and stored at −80°C. Primary keratinocytes at 50% confluence were stimulated with 100 µM final concentration of Wnt5a-mimic peptide for 18 h. Stimulation with recombinant human IFNα (PBL Biomedical Labs, order-nr 3410) was carried out analogously with 20 ng/ml final concentration. For the concentration response, final concentrations were 3, 10, 30, 100 und 300 ng/ml, respectively.

### Expression profiling

RNA was isolated and reverse transcribed to cDNA as described [16]. cDNA was fluorescence-labelled, hybridized to SkinPatho microarrays (Miltenyi Biotech), and scanned by the in-house microarray facility of the Charité Medical University, Berlin, Germany. Raw data were adjusted for red/green bias. Excel-based auto-filtering was used to exclude all transcripts lower than ten fluorescence units above the detection threshold in both the red- and green-labelled samples. P-values were determined using a standard t-test as indicated in the respective tables.

### Wnt5a transfection

Full-length human wnt5a cDNA was purchased from Origene. Five×10^5^ HaCat keratinocytes were cultivated in RPMI1640 medium and transfected in Advanced Medium (Gibco, order-nr 12491015) containing 2% FCS with 40 µg Wnt5a-pCMV-XL4 or control vector (pCDNA6.1) using CaCl_2_ precipitation as previously described for transfection of lentiviral vectors [19].

### RT-PCR

RNA was extracted using the NucleoSpin II extraction kit (Machery-Nagel, #740 955) and cDNA prepared using SuperScript II RT, RnaseOut, Oligo(dT)_12-18_ from Invitrogen. For PCR reactions, annealing temperature was uniformally 55°C. All genes were amplified for 25 cycles, except for wnt5a and IFI27 (30 cycles). Primers and conditions are detailed in Supporting Methods S1.

## Results

### Dysregulation of Wnt-signalling in hyperproliferative epidermis

Psoriasis is a skin disease characterized by hyperproliferation, as well as aberrant differentiation of epidermal keratinocytes. In the course of extensive expression profiling we previously noted that the non-canonical Wnt family member Wnt5a was greatly upregulated in psoriatic epidermis [16]. Accordingly, we carried out a more comprehensive expression analysis of Wnt-signalling molecules in psoriatic skin. As shown in table 1, only Wnt5a and Fzd6 were significantly upregulated whereas the β-catenin inhibitor ICAT was downregulated (shown in bold print), in confirmation of previous findings [18], [20].

**Table 1:** Expression of Wnt-related genes in psoriasis.^1^

Gene	Location	p-value	fold change
Wnt proteins			
WNT1	12q13	-	n.d.
WNT2	7q31	0.83	1.07
WNT2B	1p13	-	n.d.
WNT4	1p36.23-p35.1	0.78	−1.06
**WNT5A**	3p21-p14	0.0004	4.54
WNT6	2q35	0.017	1.79
WNT7A	3p25	-	n.d.
WNT8B	10q24	-	n.d.
WNT10B	12q13	0.24	−1.37
WNT11	11q13.5	0.14	−1.53
Frizzled-related proteins			
FZD1	7q21	0.18	−1.42
FZD2	17q21.1	-	n.d.
FZD3	17q21.1	0.39	−1.15
FZD4	11q14.2	0.75	1.15
FZD5	2q33-q34	0.18	1.34
**FZD6**	8q22.3-q23.1	0.027	1.99
FZD7	2q33	0.056	−1.58
FZD9	7q11.23	-	n.d.
FRZB	2qter	0.45	−1.41
SFRP1	8p12-p11.1	0.16	−1.49
SFRP4	7p14.1	0.10	−1.45
Other membrane receptors			
LRP5	11q13.4	0.79	−1.08
LRP6	12p11-p13	0.37	1.07
LRP1	12q13-q14	0.27	−1.30
ROR2	9q22	-	n.d.
RYK	3q22	0.22	−1.38
Wnt-inhibitors			
WIF1	12q13.13	0.24	−1.58
DKK1	10q11.2	0.39	−1.47
DKK4	8p11.2-11.1	0.37	1.12
Downstream effectors			
DVL3	3q27	0.99	−1.01
GSK3B	3q13.3	0.09	1.26
APC	5q21-q22	-	n.d.
CTNNB1	3p21	0.95	1.02
**CTNNBIP1**	1p36.22	0.006	−2.42

1 Expression of wnt-signalling related molecules was analyzed using the Affymetrix U95A microarray. Data shown represent fold-changes in psoriatic lesional vs. non-lesional skin of n = 5 independent donors, as detailed in [16]. **Bold**-print: p<0.05 and fold-change >1.5; n.d.: not detected.

### Expression of Wnt5a and Fzd receptors in adult human epidermis

**Figure 1:**
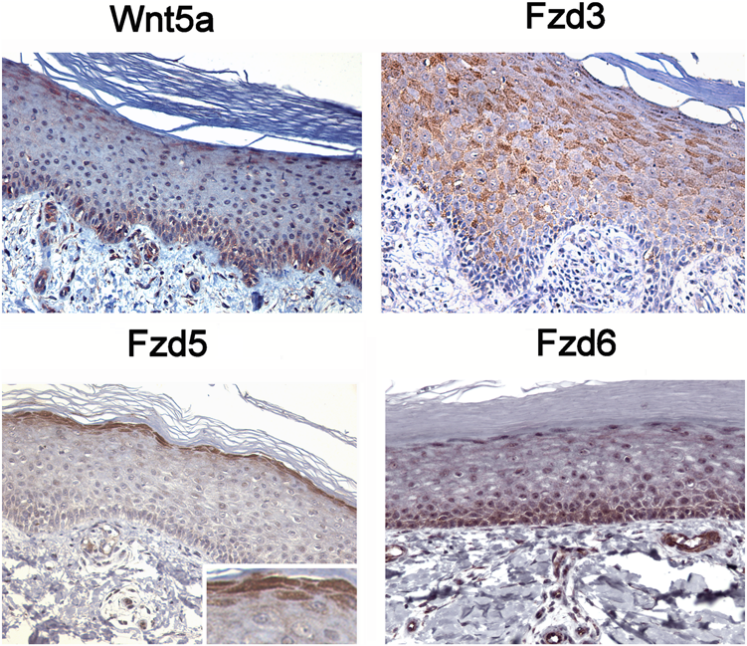
Expression of Wnt5a, Fzd3, Fzd5, and Fzd6 in adult human epidermis. Immunohistochemistry of paraffin-embedded skin samples was performed as detailed in Methods. Panels shown are at 200× magnification.

**Figure 2:**
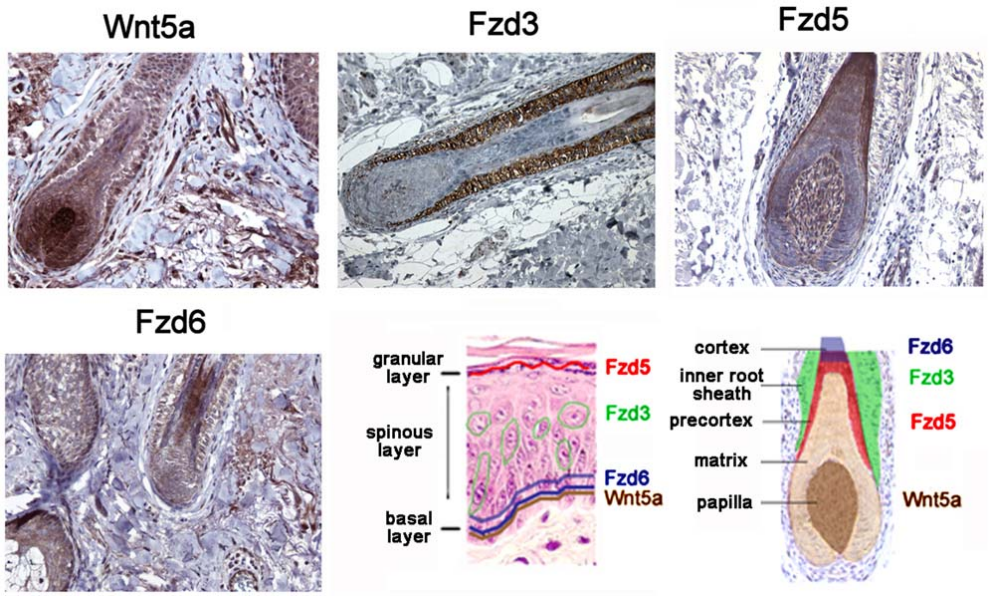
Expression of Wnt5a and Fzd3, Fzd5, and Fzd6 in adult human hair follicles. Immunohistochemistry of paraffin-embedded skin samples was performed as detailed in Methods. Panels shown are at 200× magnification. The hair follicle schematic for the cartoon on lower right was adapted from [11].

Before analyzing Wnt5a expression in psoriasis on the protein level, we first performed a detailed immunohistochemical expression analysis in normal adult human skin which has not been published to date. We included Fzd3 and Fzd5 in this analysis, since these have been reported to bind Wnt5a, as well as Fzd6, as it was found upregulated in psoriasis (table 1) and squamous cell carcinoma [18]. The epidermal expression profiles are shown in figure 1. Wnt5a is mainly expressed in the basal layer and few cells in the upper spinous and granular layers. Fzd3 was found to be strictly confined to the spinous layer in the epidermis and was the only receptor exhibiting a non-homogeneous patchy expression pattern such that strongly positive cells are intersperced with Fzd3-negative cells in a mosaic-like pattern. Fzd5 expression is strictly limited to the granular layer (magnified inset) whereas Fzd6, similar to Wnt5a, is mainly found in the basal layer, corresponding to its distribution in murine skin [21] (fig. 1e therein). In the hair follicle, Fzd 3 is mainly expressed in the inner root sheath, Fzd 5 in the precortex, and Wnt5a in the papilla (fig. 2). These findings are consistent with previous data obtained in postnatal murine skin [11], [22], thereby also confirming the specificity of the antibodies employed here. In addition, we also observe Fzd6 expression in the cortex. Taken together, the highly compartmentalized distribution of Wnt5a and Fzd isoforms in adult human epidermis is reminiscent of their zonal restriction in hair follicles (see fig. 2, lower right), strongly suggesting that Wnt signalling regulates differentiation not only in hair follicles, but also in the epidermis.

### Expression of Wnt5a and receptors in psoriasis

**Figure 3:**
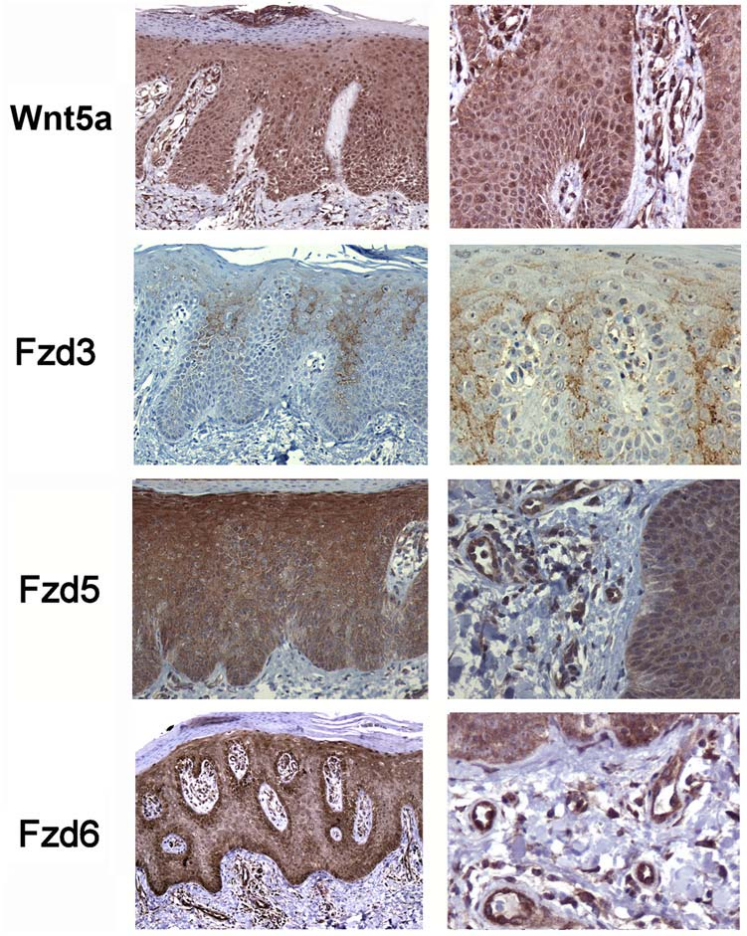
Expression of Wnt5a and Fzd proteins in psoriasis. (a) Immunohistochemistry of Wnt5a was performed as detailed in Methods. Panels on left are 200×, panels on right are at 400× magnification. Wnt5a staining was virtually identical in n = 12 samples from independent patients.

We next characterized the expression of Wnt5a, Fzd3, Fzd5, Fzd6 in psoriasis lesions which are characterized by hyperproliferation and dysregulated differentiation. As shown in figure 3, Wnt5a was found to be overexpressed throughout the entire spinous layer. Moreover, as shown in the top panel on the right, activated endothelials cells and dermal fibroblasts are strongly positive for Wnt5a. Finally, the neutrophil aggregates in the stratum corneum, a hallmark of psoriasis, also express Wnt5a (arrow, top left panel). While Fzd3 shows an expression pattern qualitatively unchanged to normal epidermis, Fzd5 is shifted from its exclusive expression in the granular layer towards overexpression in all epidermal layers as well as expression in the dermal endothelia (high power image on right). Fzd6 is also heavily overexpressed as well as in the endothelia but retains the same relative expression level as in normal epidermis (strongest staining in the basal layer). Taken together, Wnt5a, Fzd5, and Fzd6 are strongly upregulated in psoriasis. In addition, Wnt5a and Fzd5 exhibit an altered qualitative distribution.

### Overexpression of Wnt5a in keratinocytes from psoriasis patients in vitro

**Figure 4:**
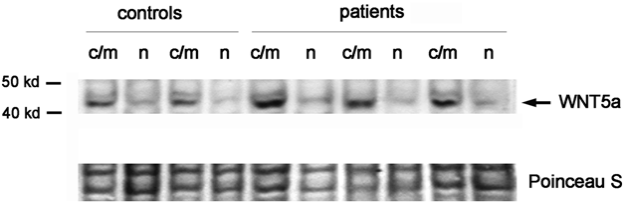
Overexpression of Wnt5a in psoriatic keratinocytes in vitro. Keratinocytes from non-affected skin from psoriasis patients or healthy control skin were expanded in vitro for 14 days, fractionated in nuclear (n) or cytoplasmic/membrane fractions (c/m), and subjected to western blot, as detailed in Methods. 50 µg of protein were loaded per lane. The bottom panel shows the Poinceau-S stain of the blot verifying even protein loading. * = non-specific band also visible in the Poinceau S stain.

We next sought to clarify whether any of the observed changes represent a cell-autonomous feature of the hyperproliferative keratincoytes in psoriasis patients, i.e. whether they can be detected in vitro in the absence of the inflammatory micro-environment. To this end, we expanded keratinocytes from *non*-lesional skin of psoriasis patients, or healthy control patients, *in vitro* and performed microarray-based expression profliling. As shown in table 2, Wnt5a was found significantly upregulated on the mRNA level in keratinocytes derived from psoriasis patients. This was not the case for Fzd3 and Fzd5 (Fzd6 was not represented on the Skin-Patho microarray used for this experiment). By western blot, we were able to confirm that Wnt5a protein is indeed overexpressed in primary human keratinocytes expanded from non-lesional skin of psoriasis patients (fig. 4). Although the low sample number precludes a definite statement, these data suggest that upregulation of Wnt5a may constitute of cell-autonomous feature of psoriatic keratinocytes. Regardless of this notion, the clear difference in Wnt5a expression between the psoriasis- and control-derived keratinocytes characterized here is suitable to study effects of endogenous Wnt5a overexpression (see below).

**Table 2:** Genes with altered expression in cultured psoriatic keratinocytes.^1^

	FC in vitro^2^	p-value^3^	FC skin^4^	p-value skin^4^
Increased expression				
KYNURENINASE	5.5	0.003	3.4	0.06
COL3A1	3.2	0.006	2.9	0.018
COL5A2	3.1	0.006	n.s.	n.s.
COL6A1	3.0	0.000	n.s.	n.s.
WNT5A	2.9	0.003	4.5	0.0004
COL1A1	2.5	0.009	n.s.	n.s.
CXCL1 (GRO1)	2.0	0.008	n.s.	n.t.
MMP2	1.8	0.008	n.s.	n.s.
Decreased expression				
ARHE (Rho8)	0.6	0.002	3.3	0.005
PLK2 (SNK)	0.5	0.010	2.4	0.002

1 Keratinocytes from non-lesional skin of psoriasis patients (n = 3) or normal control skin (n = 4) were expanded in vitro and cultured for appr. 14 days, followed by expression profiling using the PIQUOR Skin-Patho microarray, as detailed in methods.

2 Fold change (relative expression levels in keratinocytes from psoriasis patients vs. controls).

3 Statistical significance, as determined by a non-paired two sided t-test.

4 Overexpression in lesional vs. non-lesional psoriatic skin, as previously reported [16].

### Wnt5a induces type I interferon target genes

**Figure 5:**
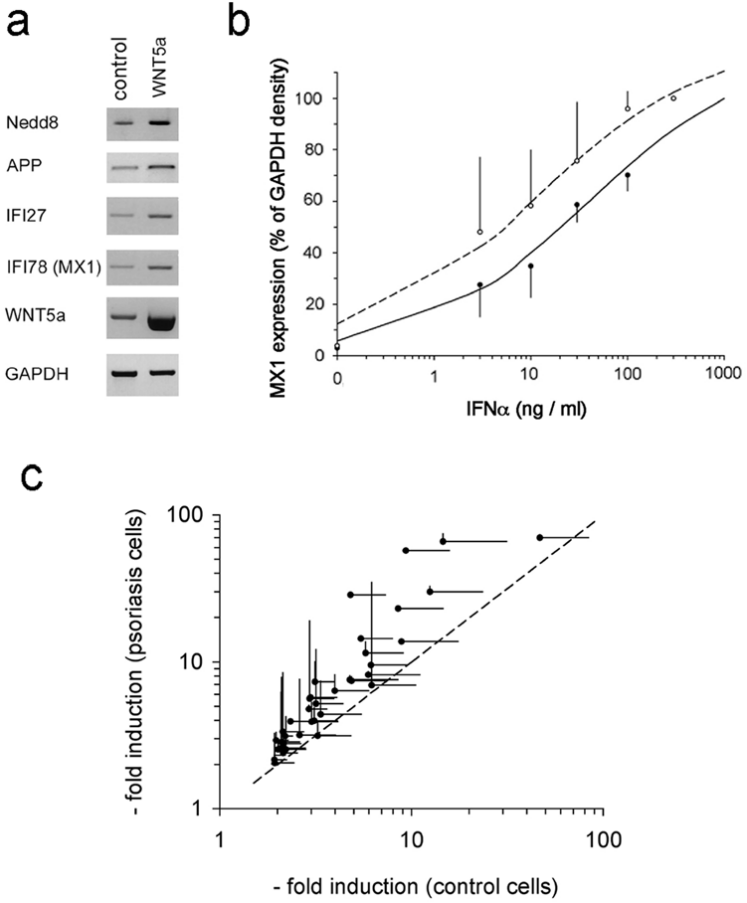
Wnt5a induces type 1 IFN target genes and causes IFN hypersensitivity. (a) HaCat keratinocytes were transfected with full length Wnt5a cDNA or control vector (pCDNA6.1). 12 h after transfection, the expression of nedd8, MX1, IFI-27, and APP was determined by RT-PCR, as described in Methods. The expression of Wnt5a was also determined to verify overexpression. (b) HaCat keratinocytes transfected with full-length Wnt5a cDNA (open symbols) or control vector (closed symbols) and stimulated with the indicated concentrations of IFNα for 18 h. The expression of IFI27 was then assessed using RT-PCR and quantified densitometrically. The data shown represent mean±s.d. of two independent experiments. (c) Keratinocytes from non-lesional psoriatic skin with elevated endogenous Wnt5a levels or from healthy control skin were expanded in vitro for 14 days and stimulated for 18 h with 20 ng/ml IFNα. Global gene expression was determined using the piquor skin-patho microarray. Plotted are the –fold change (IFNα vs. basal expression) of all genes (41 of 1133) exhibiting a ≥2-fold induction by IFNα. The mean fold-changes of cells from n = 3 controls are plotted along the x-axis, the mean fold-change of cells from n = 4 cells are plotted along the y-axis. Dots represent mean values, horizontal lines represent s.d. among the controls, vertical lines represent s.d. among cells from psoriasis patients. The dashed line marks the theoretical equal magnitude of gene induction.

In order to screen for cellular signalling pathways possibly affected by Wnt5a in keratinocytes, we stimulated keratinocytes in vitro with a formylated wnt5a-derived hexapeptide shown to mimic the effect of full-length wnt5a on adherence and motility in vivo and in vitro [5], [23] and compared global gene expression between keratinocytes stimulated with Wnt5a-mimic peptide and control peptide. We performed this experiment both with keratinocytes from psoriasis patients and from control patients. Since there were no significant differences in the effect of the Wnt5a-mimic peptide on keratinocytes from psoriasis patients and controls (not shown), both groups were analyzed together. Of the 1133 transcripts represented on the Piquor SkinPatho microarray, only four exhibited a greater than 1.2-fold change: nedd8, MX1 (IFI-78), IFI-27, and APP (table 3). Both MX1 and IFI-27 are type 1 interferon target genes previously also found to be upregulated in psoriatic skin lesions [16]. Induction of all four target genes could subsequently be verified by RT-PCR of HaCat keratinocytes transfected either with full-length Wnt5a cDNA or control vector (fig. 5a). Importantly, type 1 IFN genes themselves (α or β) were not induced by Wnt5a (not shown).

**Table 3:** Genes induced in keratinocytes by Wnt5a in vitro.^1^

Gene ID	fold change			p-value^2^
	Psoriasis	Controls	Total	
NEDD8	1.34	1.35	1.35	2×10^−7^
IFI-78 (MX1)	1.53	1.16	1.35	0.14
IFI27	1.52	1.12	1.32	0.11
APP	1.41	1.14	1.28	0.002

1 Primary keratinocytes expanded from healthy control donors (n = 3) or psoriasis patients (n = 3) were cultivated in the presence or absence of Wnt5a mimic peptide [5] or control peptide for 48 h and gene expression subsequently analyzed as detailed in Methods. Total fold-changes shown represent the average across all six samples (wnt5a vs. control peptide).

2 As determined by a two-sided paired t-test.

### Wnt5a causes hypersensitivity toward type 1 interferon

Since the magnitude of mRNA upregulation was rather modest for an IFN target gene, we hypothesized that a possible function of Wnt5a might be to „prime“ cells for synergistic induction by type 1 IFN. To test this hypothesis, we transfected HaCat keratinocytes either with full-length Wnt5a cDNA or control vector. Transfected cells were then exposed to increasing concentrations of IFNα and expression of the IFNα target gene MX1 determined by PCR. As shown in figure 5b, concurrent overexpression of Wnt5a caused a left shift in the concentration response curve to all IFNα where the calculated apparent EC-50 shifted from 22 ng/ml to 6 ng/ml, suggesting that overexpression of Wnt5a indeed increases sensitivity toward IFNα. This observation predicts that low concentrations of IFNα should cause a greater magnitude of IFN target gene induction in cells with increased Wnt5a expression. To test this, we compared the response to type 1 IFN between primary epidermal keratinocytes from psoriasis patients harbouring increased endogenous Wnt5a protein levels (see above, fig. 4) to keratinocytes from control probands. We stimulated these cells with 20 ng/ml of IFNα, a concentration which, according to the data presented above, would be expected to cause a higher magnitude of IFNα target gene induction in keratinocytes with endogenous Wnt5a overexpression. After 18 h of stimulation, global gene expression was analyzed using the Piquor SkinPatho microarray. Figure 5c shows all transcripts (41 of 1133) present on the array which were induced at least a two-fold by IFNα. The induction level is expressed as mean fold-change of all control keratinocytes (n = 4, along the x-axis) vs. mean fold-change of all psoriasis keratinocytes (n = 3, along the y-axis). Strikingly, all 41 IFN-responsive target genes are induced to a higher level in the keratinocytes harbouring higher Wnt5a levels, corresponding to a statistically highly significant 1.8±1.1-fold relative hyper-induction across all target genes (p = 0.001). These data confirm that hypersensitivity toward IFNα, i.e. increased IFN target gene induction at low IFN concentrations, is a general feature detectable for all target genes in primary cells with endogenous Wnt5a upregulation.

### APP and nedd8 are synergistically induced by type 1 IFN and Wnt5a

**Figure 6:**
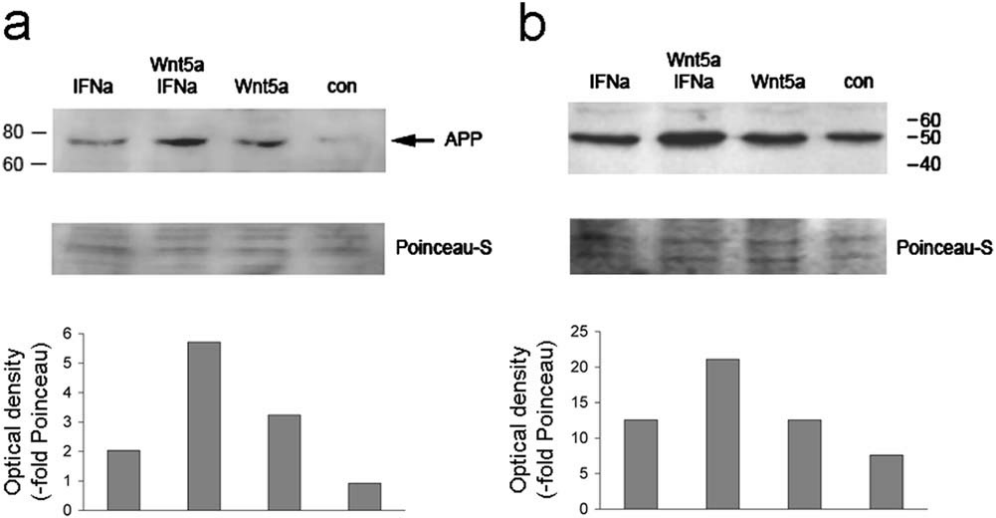
Synergistic induction of APP and nedd8 by Wnt5a and IFNα. (a) HaCat cells were transfected either with control vector or Wnt5a, followed by incubation in the absence or presence of 20 ng/ml IFNα for 18 h. Whole cell lysates were subjected to SDS-PAGE and blotted with anti-APP. The visible band migrating at appr. 70 kd is consistent with β-secretase-cleaved sAPP. Poinceau-S stain of the membrane is shown as loading control. (b) HaCat cells were transfected and stimulated with IFNα as in (a), followed by preparation of nuclear extracts, SDS-PAGE, and western blot using anti-nedd8.

Apart from MX1 and IFI-27, the only transcripts induced by Wnt5a in keratinocytes were APP and nedd8 (see above, table 3 and figure 5). Wnt5a alone showed only a modest induction of both genes in keratinocytes. Likewise, stimulation by IFNα alone only caused a slight upregulation of either gene (1.4±0.6 fold and 1.6±0.4 fold for APP and nedd8, respectively). Therefore, we hypothesized that these genes, too, might be subject to synergistic induction by Wnt5a and type 1 IFN. To test this hypothesis we transfected HaCat keratinocytes either with control vector or full-length Wnt5a, and cultivated the transfected cells either in the presence or absence of 20 ng/ml IFNα. As shown in fig. 6a, a specific band at appr. 70 kd, corresponding to soluble APP, was induced upon either Wnt5a or IFNα stimulation, but more strongly upon co-stimulation with both factors. Synergistic induction of soluble APP is also evident upon densitometric quantification of the blot, as shown on the bottom of the figure. Nedd8 is a ubiquitin-like peptide covalently linked to a number of target proteins. In nuclear extracts of HaCat cells, a nedd8-reactive protein migrating at appr. 50 kd was present in control cells, but more abundant after either Wnt5a-transfection or IFNα stimulation and clearly synergistically induced by both factors (fig. 6b), which is also seen upon densitometric quantification. In conjunction with the transcriptional induction of nedd8 by IFNα as well as Wnt5a seen on the mRNA level (figure 4a), these data strongly suggest that nedd8 is synergistically induced by Wnt5a and IFNα. Taken together, APP (previously implicated in psoriasis pathogenesis) and nedd8 represent novel interferon targets induced significantly by the concurrent action of Wnt5a and IFNα.

## Discussion

We here show that Wnt5a and its putative receptor Fzd5 are overexpressed in psoriasis, that Wnt5a increases the sensitivity of keratinocytes towards type 1 interferon, and that nedd8 and APP are synergistically induced by Wnt5a and IFNα.

The unexpected finding that Wnt5a and fzd3, fzd5, and fzd6 proteins are highly compartmentalized in normal adult human epidermis, reminiscent of the distribution of wnt signalling components in the hair follicle, raises the question of the role of this system in the dynamics of keratinocyte turnover in the skin. Of all relevant classical knock-out phenotypes, only Fzd6-deficient mice survive post term with intact epidermis except for abnormal hair whorls [21], suggesting that its physiological presence in the basal layer is dispensable. Therefore, inducible loss- or gain-of-function approaches will be required to delineate the functional role of wnt signalling in post-natal skin.

The marked overexpression of Wnt5a and Fzd5 in psoriasis suggest that this ligand-receptor pair may actively drive the chronic inflammatory and hyperproliferative nature of this phenotype. This idea receives further support from a large body of circumstantial evidence. First, WNT5a induces IL12 [6], which is central to psoriasis, as indicated by the therapeutic efficacy of antibodies targeted at IL12p40 and by the genetic association of IL12 with psoriasis [24], [25]. Second, Wnt5a is itself strongly induced by STAT3 [26], thereby acting as a potential effector molecule for the psoriasis-like skin disease seen in STAT3-overexpressing transgenic mice [27]. Third, WNT5a induces epithelial differentiation and stimulates endothelial cell proliferation, both important elements of psoriasis. Fourth, Wnt5a is upregulated in wound healing [28] and psoriasis lesions can classically be triggered by skin wounding. Fifth, our data show that dermal fibroblasts in psoriasis are strongly positive for Wnt5a and activated fibroblasts from rheumatoid arthritis are synthesize inflammatory cytokines, most notably IL15, subsequent to Wnt5a/Fzd5 signalling [29]. Sixth, Wnt5a derived from endothelia signals through Fzd5 to induce the translocation of NFAT and subsequent induction of IL-2 in T cells [10]. This effect may be highly relevant, since (i) continued T cell activation also represents a central element in psoriasis [30], and (ii) Wnt5a-mediated T cell activation can be elicited by lithium which is one of the most potent clinical triggers of psoriasis flares [31]. Finally, and most importantly, WNT5a induces IFN hypersensitivity (see below), a central hallmark of psoriasis.

The relationship between Wnt5a and β-catenin signalling in psoriasis as well as physiological epidermal turn-over is unclear. Supra-basal accumulation of nuclear β-catenin in psoriasis has been reported [32]. Moreover, we here report down-regulation of two inhibitors of canonical wnt-signalling in psoriasis, ICAT (CTNNBIP1) and WIF (table 1). Furthermore, the psoriasis-inducing effect of lithium mentioned above may also involve GSKβ-inhibition and β-catenin activation in vivo. Thus, Wnt5a and β-catenin activation may occur concurrently in psoriasis. Indeed, retroviral overexpression of Wnt5a after wounding in mice did not antagonize nuclear translocation of β-catenin [28].

Wnt5a signalling in psoriasis may also be related to the activation of the nuclear hormone receptor PPARδ which we have previously identified as a central mediator in psoriasis. Thus, Wnt5a may synergize with IFNα to induce the expression of PPARδ in activated T lymphocytes [29]. Moreover, PPARδ stimulates keratinocyte proliferation and in several biological processes acts by antagonizing another PPAR isoform, PPARγ [16]. Wnt5a also represses PPARγ transactivation thereby synergizing with PPARδ functionally [33]. Conversally, PPARγ inhibits STAT3 [34] which, when overexpressed, induces Wnt5a and causes a psoriasis-like phenotype in vivo [27].

The molecular mechanism of Wnt5a/IFNα crosstalk is elusive as no data exist on the effect of frizzled-mediated signalling on downstream effectors of IFN signalling. Our results do suggest that Fzd5 may be the responsible molecule at the receptor level and antibody-mediated neutralization studies will be able to clarify this issue. Moreoever, our data do not show a direct transcriptional upregulation of IFN genes or IFN receptors (data not shown) by Wnt5a activation. Down-stream of the membrane receptor, it is less clear at which point Fzd-mediated signals interact with IFN signalling relayers [35]. The present data should certainly prompt a more detailed analyses of the effect of Wnt5a on nuclear translocation and phosphorylation of factor involved in IFN signal transduction.

We here identify the APP amyloid precursor protein and the ubiquitin-like molecule nedd8 as bona fide targets of synergistic IFNα/Wnt5a induction. APP is upregulated and re-distributed in psoriasis lesions strikingly similar to Wnt5a and APP stimulates both proliferation and mobility of keratinocytes [36], [37]. Furthermore, processed amyloid β induces transcription of kynureninase, which we found to be strongly upregulated in psoriasis in vivo, as well as in psoriasis-derived cells in vitro (table 2), and which can elicit an inflammatory skin reaction in vivo [38]. Therefore, the induction of APP by Wnt5a/IFN may contribute to increased keratinocyte turnover and migration in psoriasis. As for nedd8, further studies must clarify which target proteins are neddylated by concurrent Wnt5a/IFN stimulation and whether these are involved in epidermal dynamics. However, the results presented here clearly show that, in addition to established IFN target genes, another class of targets exists which are synergistically induced by Wnt5a/IFNα. Thus, screening for such targets in other cell types such as T cells, endothelial cells, or fibroblasts, would delineate the profile of innate immune activation elicited by Wnt5a.

In conclusion, we here demonstrate compartmentalized distribution of Wnt-signalling components in adult skin, up-regulation of Wnt5a in hyperproliferative and differentiation-deficient epidermis, and identify sensitization for interferon as a novel function of Wnt signalling.

## Supporting Information

Supporting Methods S1 (0.06 MB DOC)
