# Supplementary material for: Wnt5a Exhibits Layer-Specific Expression in Adult Skin, Is Upregulated in Psoriasis, and Synergizes with Type 1 Interferon
Source: PLoS One. 2009 Apr 28;4(4):e5354. doi: 10.1371/journal.pone.0005354 (PMC2670517; doi:10.1371/journal.pone.0005354)
Supplement: Supporting Methods S1 — (0.06 MB DOC) [file pone.0005354.s001.doc]

Supplement to „*Wnt5a exhibits layer-specific expression in adult skin, is upregulated in psoriasis, and synergizes with type 1 interferon*“

**Immunohistochemistry protocol:**

Sections from paraffin embedded tissue (nominally 4 microns thick) were cut onto superfrost® plus slides (VWR International Ltd) and dried for 1 hr at 60 °C before being de-paraffinised in Histoclear (National Diagnostics) and then rehydrated through a graded alcohol series. 10 mM Citric acid buffer, pH 6.0 was used as standard microwave-based antigen retrieval methods. Sections were microwaved in a pressure cooker for 15 min before being immunostained on a DAKO autostainer using Vectastain® ABC kits (Vector Labs) according to the manufacturer’s protocol. Briefly, sections were blocked in either normal goat, rabbit or horse serum containing 10%(v/v) from stock avidin solution (Vector Labs) for 20 min followed by 1 hr incubation with primary antibody including 10%(v/v) from stock biotin solution (Vector Labs) to reduce non-specific background staining. Primary antibodies are described in Material and Methods. Sections were incubated with either biotinylated anti-rabbit or anti-goat (for polyclonal antibodies) or anti-mouse (for monoclonal antibodies) antibody for 30 min followed by Vectastain® Elite ABC reagent for another 30 min. Liquid Diaminobenzidine (DAB) (DAKO) was applied for 5 min and sections were counterstained with Mayer’s haematoxylin. Between each immunostaining step, slides were washed briefly in Tris buffered saline (TBS) buffer, pH 7.6. Sections known to stain positively were included in each batch and negative controls were prepared by replacing the primary antibody with TBS buffer.

**PCR primers and conditions:**

Annealing temperature was uniformally 55 ºC. PCR primers and cycle numbers used are listed below.

| Name | Forward Primer | Reverse Primer | Cycle number |
| --- | --- | --- | --- |
| GAPDH | gtcagtggtggacctgacct | aggggtctacatggcaactg | 25 |
| Wnt-5a | acacctctttccaaacaggcc | ggattgttaaactcaactctc | 30 |
| Nedd8 | ggtgttggctctgagaggag | agagagggaagcacacagga | 25 |
| APP | cacagagagaaccaccagca | acatccgccgtaaaagaatg | 25 |
| IFI27/ ISG12 | gcgggcattttaactgacat | cccacggagacagaaaggta | 30 |
| IFI78/ MX1 | gtgcattgcagaaggtcaga | ttcaggagccagctgtaggt | 25 |

**References**

Hung BS, Wang XQ, Cam GR, Rothnagel JA (2001) Characterization of mouse Frizzled-3 expression in hair follicle development and identification of the human homolog in keratinocytes. *The Journal of investigative dermatology* **116**:940-946.

Reddy ST, Andl T, Lu MM, Morrisey EE, Millar SE (2004) Expression of Frizzled genes in developing and postnatal hair follicles. *The Journal of investigative dermatology* **123**:275-282.

Romanowska M, al Yacoub N, Seidel H, Donandt S, Gerken H, Phillip S, et al. (2008) PPARdelta enhances keratinocyte proliferation in psoriasis and induces heparin-binding EGF-like growth factor. *The Journal of Investigative Dermatology* **128**:110-124.
